# SoftKiller, a particle-level pileup removal method

**DOI:** 10.1140/epjc/s10052-015-3267-2

**Published:** 2015-02-06

**Authors:** Matteo Cacciari, Gavin P. Salam, Gregory Soyez

**Affiliations:** 1 Université Paris Diderot, Paris, France; 2 Sorbonne Universités, UPMC Univ Paris 06, UMR 7589, LPTHE, 75005 Paris, France; 3 CNRS, UMR 7589, LPTHE, 75005 Paris, France; 4 CERN, PH-TH, 1211 Geneva 23, Switzerland; 5 IPhT, CEA Saclay, CNRS URA 2306, 91191 Gif-sur-Yvette Cedex, France

## Abstract

Existing widely used pileup removal approaches correct the momenta of individual jets. In this article we introduce an event-level, particle-based pileup correction procedure, SoftKiller. It removes the softest particles in an event, up to a transverse momentum threshold that is determined dynamically on an event-by-event basis. In simulations, this simple procedure appears to be reasonably robust and brings superior jet resolution performance compared to existing jet-based approaches. It is also nearly two orders of magnitude faster than methods based on jet areas.

## Introduction

At high-luminosity hadron colliders such as CERN’s Large Hadron Collider (LHC), an issue that has an impact on many analyses is pileup, the superposition of multiple proton–proton collisions at each bunch crossing. Pileup affects a range of observables, such as jet momenta and shapes, missing transverse energy and lepton and photon isolation. In the specific case of jets, it can add tens of GeV to a jet’s transverse momentum and significantly worsens the resolution for reconstructing the jet momentum. In the coming years the LHC will move towards higher luminosity running, ultimately increasing pileup by up to a factor of ten for the high-luminosity LHC [1]. The experiments’ ability to mitigate pileup’s adverse effects will therefore become increasingly crucial to fully exploit the LHC data, especially at low and moderate momentum scales, for example in studies of the Higgs sector.

Some approaches to reducing the impact of pileup are deeply rooted in experimental reconstruction procedures. For example, charged hadron subtraction (CHS) in the context of particle flow [2] exploits detectors’ ability to identify whether a given charged track is from a pileup vertex or not. Other aspects of pileup mitigation are largely independent of the experimental details: for example both ATLAS and CMS [3, 4] rely on the area–median approach [5, 6], which makes a global estimate for the transverse-momentum-flow density, $\rho$, and then applies a correction to each jet in proportion to its area.

In this article, we introduce and study a new generic pileup removal method. Instead of correcting individual jets, it corrects for pileup at the level of particles. Such a method should make a guess, for each particle in an event, as to whether it comes from pileup or from the hard collision of interest. Particles deemed to be from pileup are simply discarded, while the much smaller set of residual “hard-collision” particles are passed to the jet clustering. Event-wide particle-level subtraction, if effective, would greatly simplify pileup mitigation in advanced jet studies such as those that rely on jet substructure [7]. Even more importantly, as we shall see, it has the potential to bring significant improvements in jet resolution and computational speed. This latter characteristic makes our approach particularly appealing also for trigger-level applications.

The basis of our pileup suppression method, which we dub “SoftKiller” (SK), is that the simplest characteristic of a particle that affects whether it is likely to be from pileup or not is its transverse momentum. In other words, we will discard particles that fall below a certain transverse-momentum threshold. The key feature of the method will be its event-by-event determination of that threshold, chosen as the lowest $p_t$ value that causes $\rho$, in the median–area method, to be evaluated as zero. In a sense, this can be seen as the extreme limit of ATLAS’s approach of increasing the topoclustering noise threshold as pileup increases [8].

This approach might at first sight seem excessively naïve in its simplicity. We have also examined a range of other methods. For example, one approach involved an all-orders matrix-element analysis of events, similar in spirit to shower deconstruction [9, 10]; others involved event-wide extensions of a recent intrajet particle-level subtraction method [11] and subjet-level [12, 13] approaches (see also [14]); we have also been inspired by calorimeter [15–17] and particle-level [18] methods developed for heavy-ion collisions. Such methods and their extensions have significant potential. However, we repeatedly encountered additional complexity, for example in the form of multiple free parameters that needed fixing, without a corresponding gain in performance. Perhaps with further work those drawbacks can be alleviated, or performance can be improved. For now, we believe that it is useful to document one method that we have found to be both simple and effective.

## The SoftKiller method

The SoftKiller method involves eliminating particles below some $p_t$ cutoff, ${{p}_{t}^{\text {cut}}}$, chosen to be the minimal value that ensures that $\rho$ is zero. Here, $\rho$ is the event-wide estimate of transverse-momentum-flow density in the area–median approach [5, 6]: the event is broken into patches and $\rho$ is taken as the median, across all patches, of the transverse-momentum-flow density per unit area in rapidity-azimuth:

1 $$\begin{aligned} \rho = \underset{i \in \text {patches}}{\text {median}} \left\{ \frac{p_{ti}}{A_i}\right\} , \end{aligned}$$

where $p_{ti}$ and $A_{i}$ are, respectively, the transverse momentum and area of patch $i$. In the original formulation of the area–median method, the patches were those obtained by running inclusive $k_t$ clustering [19, 20], but subsequently it was realised that it was much faster and equally effective to use (almost) square patches of size $a\times a$ in the rapidity-azimuth plane. That will be our choice here. The use of the median ensures that hard jets do not overly bias the $\rho$ estimate (as quantified in Ref. [21]).^1^

Choosing the minimal transverse-momentum threshold, $p_t^{\text {cut}}$, that results in $\rho = 0$ is equivalent to gradually raising the $p_t$ threshold until exactly half of the patches contain no particles, which ensures that the median is zero. This is illustrated in Fig. 1. Computationally, $p_t^{\text {cut}}$ is straightforward to evaluate: one determines, for each patch $i$, the $p_t$ of the hardest particle in that patch, $p_{ti}^{\max }$ and then $p_t^\mathrm{cut}$ is given by the median of $p_{ti}^{\max }$ values:

2 $$\begin{aligned} p_t^{\text {cut}} = \underset{i \in \text {patches}}{\text {median}} \left\{ p_{ti}^{\max } \right\} . \end{aligned}$$

With this choice, half the patches will contain only particles that have $p_t < {{p}_{t}^{\text {cut}}}$. These patches will be empty after application of the $p_t$ threshold, leading to a zero result for $\rho$ as defined in Eq. (1).^2^ The computational time to evaluate ${{p}_{t}^{\text {cut}}}$ as in Eq. (2) scales linearly in the number of particles and the method should be amenable to parallel implementation.

Imposing a cut on particles’ transverse momenta eliminates most of the pileup particles, and so might reduce the fluctuations in residual pileup contamination from one point to the next within the event. However, as with other event-wide noise-reducing pileup and underlying-event mitigation approaches, notably the CMS heavy-ion method [15–17] (cf. the analysis in Appendix A.4 of Ref. [22]), the price that one pays for noise reduction is the introduction of biases. Specifically, some particles from pileup will be above $p_t^{\text {cut}}$ and so remain to contaminate the jets, inducing a net positive bias in the jet momenta. Furthermore some particles in genuine hard jets will be lost, because they are below the $p_t^{\text {cut}}$, inducing a negative bias in the jet momenta. The jet energy scale will only be correctly reproduced if these two kinds of bias are of similar size,^3^ so that they largely cancel. There will be an improvement in the jet resolution if the fluctuations in these biases are modest.

Figure 2 shows, on the left, the average $p_t^{\text {cut}}$ value, together with its standard deviation (dashed lines), as a function of the number of pileup interactions, $n_{\text {PU}}$. The event sample consists of a superposition of $n_{\text {PU}}$ zero bias on one hard dijet event, in 14 TeV proton–proton collisions, all simulated with Pythia 8 (tune 4C) [23]. The 4C tune gives reasonable agreement with a wide range of minimum-bias data, as can be seen by consulting MCPlots [24].^4^ The underlying event in the hard event has been switched off, and all particles have been made massless, maintaining their $p_t$, rapidity and azimuth.^5^ These are our default choices throughout this paper. The grid used to determine $p_t^{\text {cut}}$ has a spacing of $a \simeq 0.4$ and extends up to $|y| < 5$. One sees that $p_t^{\text {cut}}$ remains moderate, below $2~\text {GeV}$, even for pileup at the level foreseen for the high-luminosity upgrade of the LHC (HL-LHC), which is expected to reach an average (Poisson-distributed) number of pileup interactions of $\mu \simeq 140$. The right-hand plot shows the two sources of bias: the lower (solid) curves, illustrate the bias on the hard jets induced by the loss of genuine hard-event particles below $p_t^{\text {cut}}$. Jet clustering is performed with the anti-$k_t$ jet algorithm [28] with $R=0.4$, as implemented in a development version of FastJet 3.1 [29, 30].^6^ The three line colours correspond to different jet $p_t$ ranges. The loss has some dependence on the jet $p_t$ itself, notably for higher values of $p_t^{\text {cut}}$.^7^ In particular it grows in absolute terms for larger jet $p_t$’s, though it decreases relative to the jet $p_t$. The positive bias from residual pileup particles (in circular patches of radius $0.4$ at rapidity $y=0$) is shown as dashed curves, for three different pileup levels. To estimate the net bias, one should choose a value for $n_{\text {PU}}$, read the average $p_t^{\text {cut}}$ from the left-hand plot, and for that $p_t^{\text {cut}}$ compare the solid curve with the dashed curve that corresponds to the given $n_{\text {PU}}$. Performing this exercise reveals that there is indeed a reasonable degree of cancellation between the positive and negative biases. Based on this observation, we can move forward with a more detailed study of the performance of the method.^8^

## SoftKiller performance

For a detailed study of the SoftKiller method, the first step is to choose the grid spacing $a$ so as to break the event into patches. The spacing $a$ is the one main free parameter of the method. The patch-size parameter^9^ is present also for area–median pileup subtraction. There the exact choice of this parameter is not too critical. The reason is that the median is quite stable when pileup levels are high: all grid cells are filled, and nearly all are dominated by pileup. However, the SoftKiller method chooses the $p_t^{\text {cut}}$ so as to obtain a nearly empty event. In this limit, the median operation becomes somewhat more sensitive to the grid spacing [21].

**Fig. 3:**
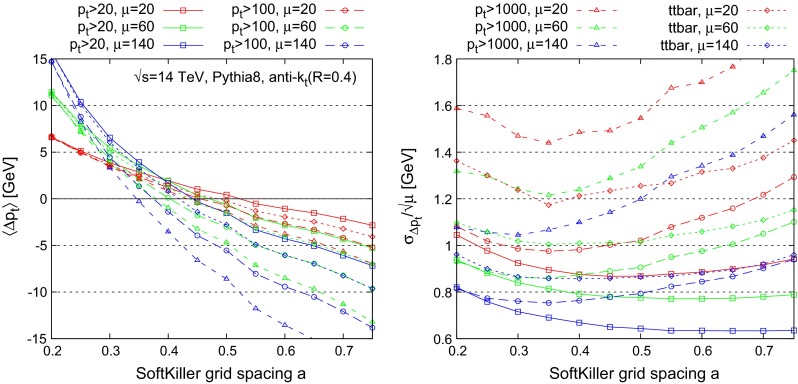
Scan of the SoftKiller performances as a function the grid-spacing parameter $a$ for different hard-event samples and three different pileup levels (Poisson-distributed with average pileup event multiplicities of $\mu =20,60,140$). *Left* Average $p_t$ shift; *right* $p_t$ shift dispersion, normalised to $\sqrt{\mu }$ for better readability. Results are given for a variety of hard processes and pileup conditions to illustrate robustness. *Curves* labelled $p_t > X$ correspond to dijet events, in which one studies only those jets that in the hard event have a transverse momentum greater than $X$. For the $t{\bar{t}}$ sample, restricted to fully hadronic decays of the top quarks, the study is limited to jets that have $p_t > 50 ~\text {GeV}$ in the hard event

Figure 3 considers a range of hard-event samples (different line styles) and pileup levels (different colours). For each, as a function of the grid spacing $a$, the left-hand plot shows the average, $\langle \Delta p_t\rangle$, of the net shift in the jet transverse momentum,

3 $$\begin{aligned} \Delta p_t = p_t^\mathrm{corrected} - p_t^\mathrm{hard}, \end{aligned}$$

while the right-hand plot shows the dispersion, $\sigma _{\Delta p_t}$, of that shift from one jet to the next, here normalised to $\sqrt{\mu }$ (right).

One sees that the average jet $p_t$ shift has significant dependence on the grid spacing $a$. However, there exists a grid spacing, in this case $a\simeq 0.4$, for which the shift is not too far from zero and not too dependent either on the hard sample choice or on the level of pileup. In most cases the absolute value of the shift is within about $2~\text {GeV}$, the only exception being for the $p_t > 1000~\text {GeV}$ dijet sample, for which the bias can reach up to $4~\text {GeV}$ for $\mu =140$. This shift is, however, still less than the typical best experimental systematic error on the jet energy scale, today of the order of $1~\%$ or slightly better [32, 33].

It is not trivial that there should be a single grid spacing that is effective across all samples and pileup levels: the fact that there is can be considered phenomenologically fortuitous. The value of the grid spacing $a$ that minimises the typical shifts is also close to the value that minimises the dispersion in the shifts.^10^ That optimal value of $a$ is not identical across event samples, and can also depend on the level of pileup. However, the dispersion at $a = 0.4$ is always close to the actual minimal attainable dispersion for a given sample. Accordingly, for most of the rest of this article, we will work with a grid spacing of $a=0.4$.^11^

**Fig. 4:**
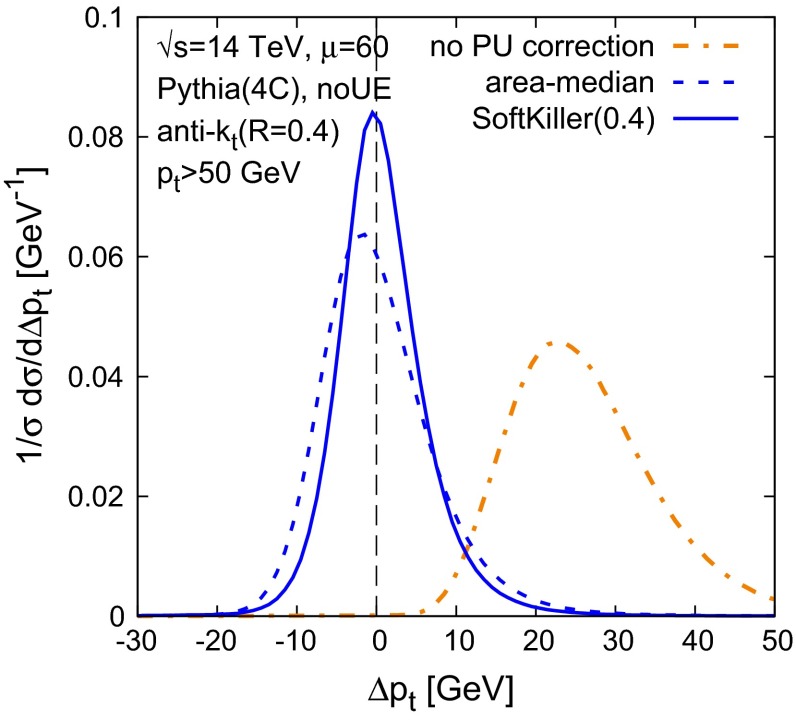
Performance of SoftKiller for 50 GeV jets and $\mu =60$ Poisson-distributed pileup events. We plot the distribution of the shift $\Delta p_t$ between the jet $p_t$ after pileup removal and the jet $p_t$ in the hard event alone. Results are compared between the area-median approach and the SoftKiller. For comparison, the (*orange*) *dash-dotted line* corresponds to the situation where no pileup corrections are applied

Next, let us compare the performance of the SoftKiller to that of area–median subtraction. Figure 4 shows the distribution of shift in $p_t$, for (hard) jets with $p_t > 50~\text {GeV}$ in a dijet sample. The average number of pileup events is $\mu = 60$, with a Poisson distribution. One sees that in the SoftKiller approach, the peak is about $30~\%$ higher than what is obtained with the area–median approach and the distribution correspondingly narrower. The peak, in this specific case, is well centred on $\Delta p_t = 0$.

**Fig. 5:**
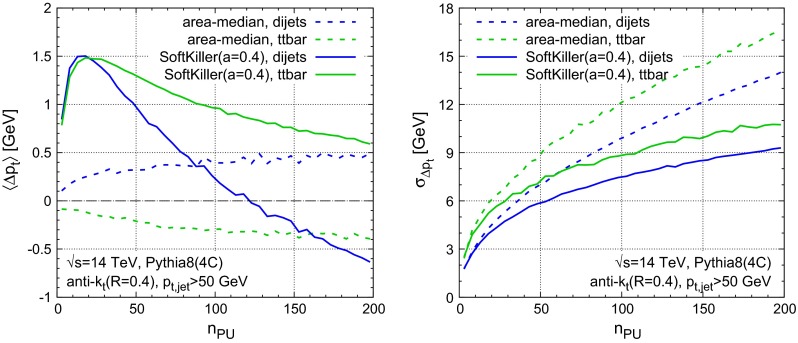
Performance of SoftKiller shown as a function of the pileup multiplicity and compared to the area-median subtraction method. *Left* The average $p_t$ shift after subtraction, compared to the original jets in the hard event. *Right* The corresponding dispersion

Figure 5 shows the shift (left) and dispersion (right) as a function of $n_{\text {PU}}$ for two different samples: the $p_t> 50~\text {GeV}$ dijet sample (in blue), as used in Fig. 4, and a hadronic $t{\bar{t}}$ sample, with a $50~\text {GeV}$ $p_t$ cut on jets (in green). Again, the figure compares the area–median (dashed) and SoftKiller results (solid). One immediately sees that the area–median approach gives a bias that is more stable as a function of $n_{\text {PU}}$. Nevertheless, the bias in the SoftKiller approach remains between about $-0.5$ and $1.5~\text {GeV}$, which is still reasonable when one considers that, experimentally, some degree of recalibration is anyway needed after area–median subtraction. As concerns the sample dependence of the shift, comparing $t{\bar{t}}$ vs. dijet, the area–median and SoftKiller methods appear to have similar systematic differences. In the case of SoftKiller, there are two main causes for the sample dependence: firstly the higher multiplicity of jets has a small effect on the choice of ${{p}_{t}^{\text {cut}}}$ and secondly the dijet sample is mostly composed of gluon-induced jets, whereas the $t{\bar{t}}$ sample is mostly composed of quark-induced jets (which have fewer soft particles and so lose less momentum when imposing a particle $p_t$ threshold). Turning to the right-hand plot, with the dispersions, one sees that the SoftKiller brings about a significant improvement compared to area–median subtraction for $n_{\text {PU}} \gtrsim 20$. The relative improvement is greatest at high pileup levels, where there is a reduction in dispersion of $30$–$35~\%$, beating the $\sqrt{n_\text {PU}}$ scaling that is characteristic of the area–median method. While the actual values of the dispersion depend a little on the sample, the benefit of the SoftKiller approach is clearly visible for both.

Figure 6 shows the shift (left) and dispersion (right) for jet $p_t$’s and jet masses, now as a function of the hard jet minimum $p_t$. Again, dashed curves correspond to area–median subtraction, while solid ones correspond to the SoftKiller results. All curves correspond to an average of $60$ pileup interactions. For the jet $p_t$ (blue curves) one sees that the area–median shift ranges from $0.5$ to $0 ~\text {GeV}$ as $p_t$ increases from $20~\text {GeV}$ to $1~\text {TeV}$, while for SK the dependence is stronger, from about $2$ to $-1~\text {GeV}$, but still reasonable. For the jet mass (green curves), again the area–median method^12^ is more stable than SK, but overall the biases are under control, at the $1$ to $2~\text {GeV}$ level. Considering the dispersions (right), one sees that SK gives a systematic improvement, across the whole range of jet $p_t$’s. In relative terms, the improvement is somewhat larger for the jet mass (${\sim }30~\%$) than for the jet $p_t$ (${\sim }20~\%$).

Figure 7 shows the actual mass spectra of $R=0.4$ jets, for two samples: a QCD dijet sample and a boosted $t{\bar{t}}$ sample. For both samples, we only considered jets with $p_t > 500~\text {GeV}$ in the hard event. One sees that SK gives slightly improved mass peaks relative to the area–median method and also avoids area–median’s spurious peak at $m=0$, which is due to events in which the squared jet mass came out negative after four-vector area subtraction and so was reset to zero. The plot also shows results from the recently proposed Constituent Subtractor method [11], using v. 1.0.0 of the corresponding code from FastJet Contrib [34]. It too performs better than area–median subtraction for the jet mass, though the improvement is not quite as large as for SK.^13^

One might ask why we concentrated on $R=0.4$ jets here, given that jet-mass studies often use large-$R$ jets. The reason is that large-$R$ jets are nearly always used in conjunction with some form of grooming, for example trimming, pruning or filtering [35–37]. Grooming reduces the large-radius jet to a collection of small-radius jets and so the large-radius groomed-jet mass is effectively a combination of the $p_t$’s and masses of one or more small-radius jets.

For the sake of completeness, let us briefly also study the SoftKiller performance for large-$R$ jets. Figure 8 shows jet-mass results for the same $t{\bar{t}}$ sample as in Fig. 7 (right), now clustered with the anti-$k_t$ algorithm with $R=1$. The left-hand plot is without grooming: one sees that SK with our default spacing of $a=0.4$ gives a jet mass that has better resolution than area–median subtraction (or the ConstituentSubtractor), but a noticeable shift, albeit one that is small compared to the effect of uncorrected pileup. That shift is associated with some residual contamination from pileup particles: in an $R=0.4$ jet, there are typically a handful of particles left from pileup, which compensate for low-$p_t$ particles lost from near the core of the jet. If one substantially increases the jet radius without applying grooming, then that balance is upset, with substantially more pileup entering the jet, while there is only slight further loss of genuine jet $p_t$. To some extent this can be addressed by using the SoftKiller with a larger grid spacing (cf. the $a=0.8$ result), which effectively increases the particle ${{p}_{t}^{\text {cut}}}$. This comes at the expense of performance on small-$R$ jets (cf. Fig. 3). An interesting, open problem is to find a simple way to remove pileup from an event such that, for a single configuration of the pileup removal procedure, one simultaneously obtains good performance on small-$R$ and large-$R$ jets.^14^

As we said above, however, large-$R$ jet masses are nearly always used in conjunction with some form of grooming. Figure 8 (right) shows that when used together with trimming [35], SoftKiller with our default $a=0.4$ choice performs well both in terms of resolution and shift.

**Fig. 9:**
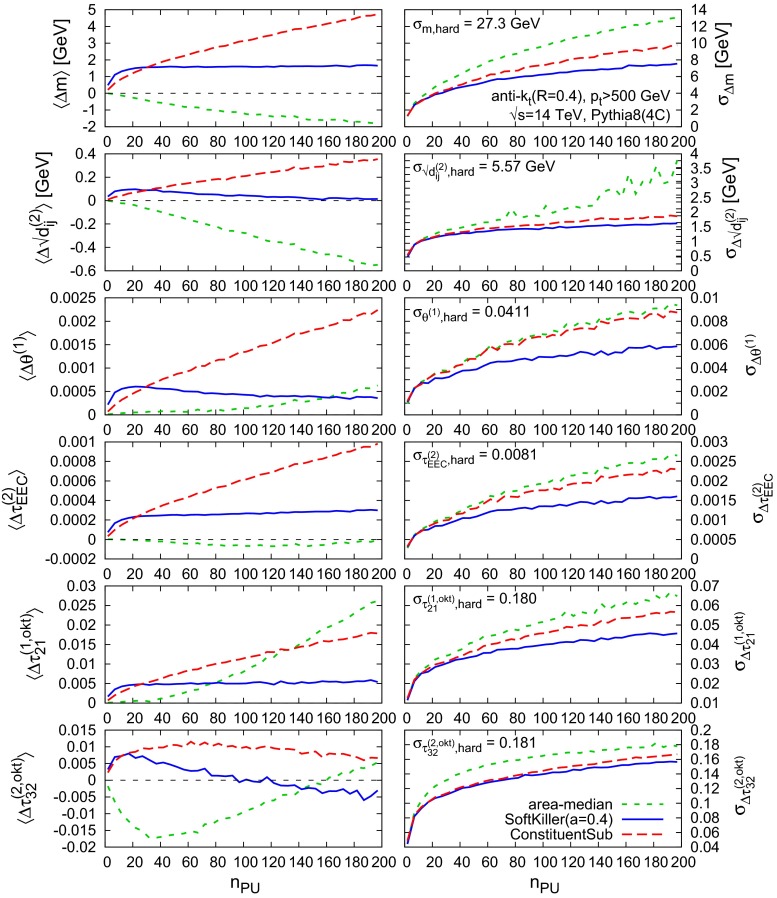
Performance of the SoftKiller on jet shapes, compared to area–median subtraction and the recently proposed Constituent Subtractor method [11]. All results are shown for dijet events with a 500 GeV $p_t$ cut on anti-$k_t$, $R=0.4$ jets. For comparison of the subtraction performance we also quote, for each observable $X$, $\sigma _{X,\mathrm{hard}}$, the dispersion of the distribution of the observable in the hard event. For $\tau _{21}$ there is the additional requirement (in the hard event) that the jet mass is above 30 GeV and for $\tau _{32}$ we further impose $\tau _{21}\ge 0.1$ (again in the hard event), so as to ensure infrared safety

Returning to $R=0.4$ jets, the final figure of this section, Fig. 9, shows average shifts (left) and dispersions (right) as a function of $n_{\text {PU}}$ for several different jet “shapes”: jet masses, $k_t$ clustering scales [19, 20], the jet width (or broadening or girth [38–40]), an energy–energy correlation moment [41] and the $\tau _{21}^{(\beta =1)}$ and $\tau _{32}^{(\beta =2)}$ N-subjettiness ratios [42, 43], using the exclusive $k_t$ axes with one-pass of minimisation. Except in the case of the jet mass (which uses “safe” area subtraction, as mentioned above), the area–median results have been obtained using the shape subtraction technique [27], as implemented in v. 1.2.0 of the GenericSubtractor in FastJet Contrib.

As regards the shifts, the SK approach is sometimes the best, other times second best. Which method fares worst depends on the precise observable. In all cases, when considering the dispersions, it is the SK that performs best, though the extent of the improvement relative to other methods depends strongly on the particular observable. Overall this figure gives us confidence that one can use the SoftKiller approach for a range of properties of small-radius jets.

## Adaptation to CHS events and calorimetric events

It is important to verify that a new pileup mitigation method works not just at particle level, but also at detector level. There are numerous subtleties in carrying out detector-level simulation, from the difficulty of correctly treating the detector response to low-$p_t$ particles, to the reproduction of actual detector reconstruction methods and calibrations, and even the determination of which observables to use as performance indicators. Here we will consider two cases: idealised charged-hadron subtraction, which simply examines the effect of discarding charged pileup particles; and simple calorimeter towers.

For events with particle flow [2] and charged-hadron subtraction (CHS), we imagine a situation in which all charged particles can be unambiguously associated either with the leading vertex or with a pileup vertex. We then apply the SK exclusively to the neutral particles, which we assume to have been measured exactly. This is almost certainly a crude approximation, however, it helps to illustrate some general features.

**Fig. 10:**
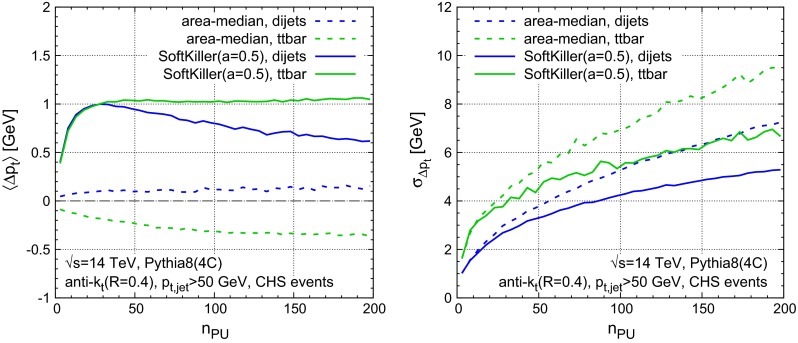
Same as Fig. 5, for events with charged-hadron subtraction (CHS). Note that the grid size used for the SoftKiller curves has been set to $a=0.5$

One important change that arises from applying SK just to the neutral particles is that there is a reduced contribution of low-$p_t$ hard-event particles. This means that for a given actual amount of pileup contamination (in terms of visible transverse momentum per unit area), one can afford to cut more aggressively, i.e. raise the $p_t^{\text {cut}}$ as compared to the full particle-level case, because for a given $p_t^{\text {cut}}$ there will be a reduced loss of hard-event particles. This can be achieved through a moderate increase in the grid spacing, to $a=0.5$. Figure 10 shows the results, with the shift (left) and dispersion (right) for the jet $p_t$ in dijet and $t{\bar{t}}$ samples. The SK method continues to bring an improvement, though that improvement is slightly more limited than in the particle-level case. We attribute this reduced improvement to the fact that SK’s greatest impact is at very high pileup, and for a given $n_\mathrm{PU}$, SK with CHS is effectively operating at lower pileup levels than without CHS. A further study with our toy CHS simulation concerns lepton isolation and is given in Appendix E.

Next let us turn to events where the particles enter calorimeter towers. Here we encounter the issue, discussed also in Appendix A, that SK is not collinear safe. While we argue there that this is not a fundamental drawback from the point of view of particle-level studies, there are issues at calorimeter level: on one hand a single particle may be divided between two calorimeter towers (we shall not attempt to simulate this, as it is very sensitive to detector details); on the other, within a given tower (say $0.1\times 0.1$) it is quite likely that for high pileup the tower may receive contributions from multiple particles. In particular, if a tower receives contributions from a hard particle with a substantial $p_t$ and additionally from pileup particles, the tower will always be above threshold, and the pileup contribution will never be removed. There are also related effects due to the fact that two pileup particles may enter the same tower. To account for the fact that towers have a finite area, we therefore adapt the SK as follows. In a first step we subtract each tower:

4 $$\begin{aligned} p_t^{\mathrm{tower},\mathrm{sub}} = \max \left( 0,p_t^\mathrm{tower} - \rho A^\mathrm{tower}\right) , \end{aligned}$$

where $\rho$ is as determined on the event prior to any correction.^15^ This in itself eliminates a significant fraction of pileup, but there remains a residual contribution from the roughly $50~\%$ of towers whose $p_t$ was larger than $\rho A^\mathrm{tower}$. We then apply the SoftKiller to the subtracted towers,

5 $$\begin{aligned} p_t^{\text {cut},\mathrm{sub}} = \underset{i \in \mathrm{patches}}{\mathrm{median}} \left\{ p_{ti}^{\mathrm{tower,sub,}\max } \right\} , \end{aligned}$$

where $p_{ti}^{{\mathrm{tower},\mathrm{sub},}\max }$ is the $p_t$, after subtraction, of the hardest tower in patch $i$, in analogy with Eq. (2). In the limit of infinite granularity, a limit similar to particle level, $A^{\mathrm{tower}}=0$. The step in Eq. (4) then has no effect and one recovers the standard SoftKiller procedure applied to particle level.

**Fig. 11:**
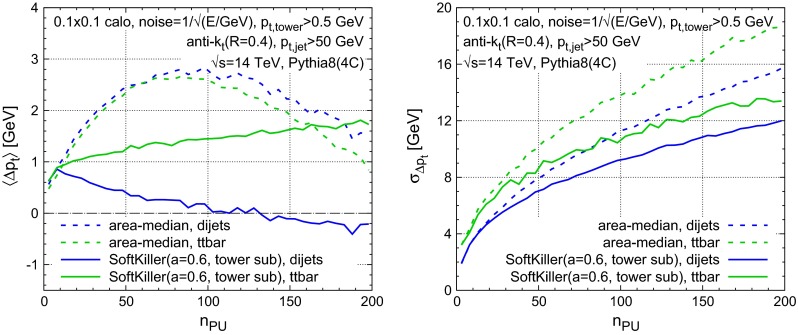
Same as Fig. 5 for events with a simple calorimeter simulation. The SoftKiller was used here with a grid spacing of $a=0.6$ and includes the tower subtraction of Eq. (4)

Results are shown in Fig. 11. The energy $E$ in each $0.1\times 0.1$ tower is taken to have Gaussian fluctuations with relative standard deviation $1/\sqrt{E/\!~\text {GeV}}$. A $p_t$ threshold of $0.5~\text {GeV}$ is applied to each tower after fluctuations. The SK grid spacing is set to $a = 0.6$. Interestingly, with a calorimeter, the area–median method starts to have significant biases, of a couple of GeV, which can be attributed to the calorimeter’s non-linear response to soft energy. The SK biases are similar in magnitude to those in Fig. 5 at particle level (note, however, the need for a different choice of grid spacing $a$). The presence of a calorimeter worsens the resolution both for area–median subtraction and SK, however, SK continues to perform better, even if the improvement relative to area–median subtraction is slightly smaller than for the particle-level results.

We have also investigated a direct application of the particle-level SoftKiller approach to calorimeter towers, i.e. without the subtraction in Eq. (4). We find that the biases were larger but still under some degree of control with an appropriate tuning of $a$, while the performance on dispersion tends to be intermediate between that of area–median subtraction and the version of SoftKiller with tower subtraction.

The above results are not intended to provide an exhaustive study of detector effects. For example, particle flow and CHS are affected by detector fluctuations, which we have ignored; purely calorimetric jet measurements are affected by the fact that calorimeter towers are of different sizes in different regions of the detector and furthermore may be combined non-trivially through topoclustering. Nevertheless, our results help illustrate that it is at least plausible that the SoftKiller approach could be adapted to a full detector environment, while retaining much of its performance advantage relative to the area–median method.

## Computing time

The computation time for the SoftKiller procedure has two components: the assignment of particles to patches, which is $\mathcal{O}\left( N\right)$, i.e. linear in the total number of particles $N$ and the determination of the median, which is $\mathcal{O}\left( P \ln P\right)$ where $P$ is the number of patches. The subsequent clustering is performed with a reduced number of particles, $M$, which, at high pileup is almost independent of the number of pileup particles in the original event. In this limit, the procedure is therefore expected to be dominated by the time to assign particles to patches, which is linear in $N$. This assignment is almost certainly amenable to being parallelised.

In studying the timing, we restrict our attention to particle-level events for simplicity. We believe that calorimeter-type extensions as described in Sect. 4 can be coded in such a way as to obtain similar (or perhaps even better) performance.

Timings are shown in Fig. 12 versus initial multiplicity (left) and versus the number of pileup vertices (right).^16^ Each plot shows the time needed to cluster the full event and the time to cluster the full event together with ghosts (as needed for area-based subtraction). It also shows the time to run the SoftKiller procedure, the time to cluster the resulting event, and the total time for SK plus clustering.

Overall, one sees nearly two orders of magnitude improvement in speed from the SK procedure, with run times per event ranging from $0.2$ to 5 ms as compared to $20$ to 300 ms for clustering with area information. At low multiplicities, the time to run SK is small compared to that needed for the subsequent clustering. As the event multiplicity increases, SK has the effect of limiting the event multiplicity to about $300$ particles, nearly independently of the level of pileup and so the clustering time saturates. However, the time to run SK grows and comes to dominate over the clustering time. Asymptotically, the total event processing time then grows linearly with the level of pileup. A significant part of that time (about $180~\text {ns}$ per particle, 75 % of the run-time at high multiplicity) is taken by the determination of the particles’ rapidity and azimuth in order to assign them to a grid cell. If the particles’ rapidity and azimuth are known before applying the SoftKiller to an event (as it would be the case e.g. for calorimeter towers), the computing time to apply the SoftKiller would be yet faster, as indicated by the dotted orange line on Fig. 12.

Because of its large speed improvement, the SoftKiller method has significant potential for pileup removal at the trigger level. Since SoftKiller returns an event with fewer particles, it will have a speed performance edge also in situations where little or no time is spent in jet-area calculations (because either Voronoi areas or fast approximate implementations are used). This can be seen in Fig. 12 by comparing the green and the solid blue curves.

## Conclusions

The SoftKiller method appears to bring significant improvements in pileup mitigation performance, in particular as concerns the jet energy resolution, whose degradation due to pileup is reduced by $20{-}30~\%$ relative to the area–median-based methods. As an example, the performance that is obtained with area–median subtraction for 70 pileup events can be extended to 140 pileup events when using SoftKiller. This sometimes comes at the price of an increase in the biases on the jet $p_t$, however, these biases still remain under control.

Since the method acts directly on an event’s particles, it automatically provides a correction for jet masses and jet shapes, and in all cases that we have studied brings a non-negligible improvement in resolution relative to the shape subtraction method, and also (albeit to a lesser extent) relative to the recently proposed Constituent Subtractor approach.

The method is also extremely fast, bringing nearly two orders of magnitude speed improvement over the area–median method for jet $p_t$’s. This can be advantageous both in time-critical applications, for example at trigger level, and in the context of fast detector simulations.

There remain a number of open questions. It would be of interest to understand, more quantitatively, why such a simple method works so well and what dictates the optimal choice of the underlying grid spacing. This might also bring insight into how to further improve the method. In particular, the method is known to have deficiencies when applied to large-$R$ ungroomed jets, which would benefit from additional study. Finally, we have illustrated that in simple detector simulations it is possible to reproduce much of the performance improvement seen at particle level, albeit at the price of a slight adaption of the method to take into account the finite angular resolution of calorimeters. These simple studies should merely be taken as indicative, and we look forward to proper validation (and possible further adaptation) taking into account full detector effects.

*Note added* As this work was being completed, we became aware of the development of another particle-level pileup removal method, PUPPI [44, 45]. Initial particle-level comparisons at the 2014 Pileup Workshop [46] suggest that both bring comparable improvements.

## Figures and Tables

**Fig. 13:**
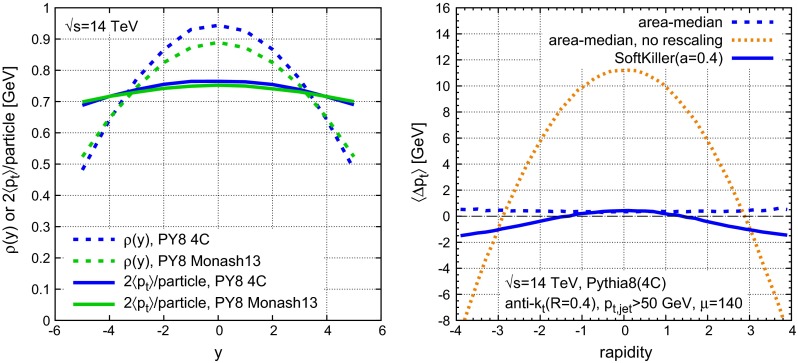
*Left* Rapidity dependence, in simulated zero-bias events, of the $p_t$ density per unit area ($\rho (y)$), and of the average $p_t$ per particle (scaled by a factor of $2$ for readability), comparing Pythia8’s 4C and Monash 2013 [47] tunes. *Right* The average shift in jet $p_t$ as a function of rapidity for the area–median method with rapidity rescaling (default in this paper), without rapidity rescaling, and for the SoftKiller as used throughout this paper, i.e. without rapidity rescaling

**Fig. 15:**
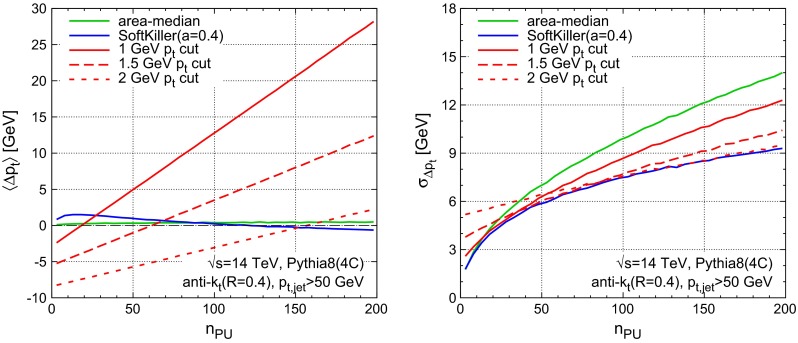
Comparison of area–median and SK results with those from a fixed $p_t$ cut. The average shift (*left*) and its dispersion (*right*) are shown as a function of $n_\mathrm{PU}$ for dijet events, with a hard jet cut of $p_t > 50~\text {GeV}$

**Fig. 16:**
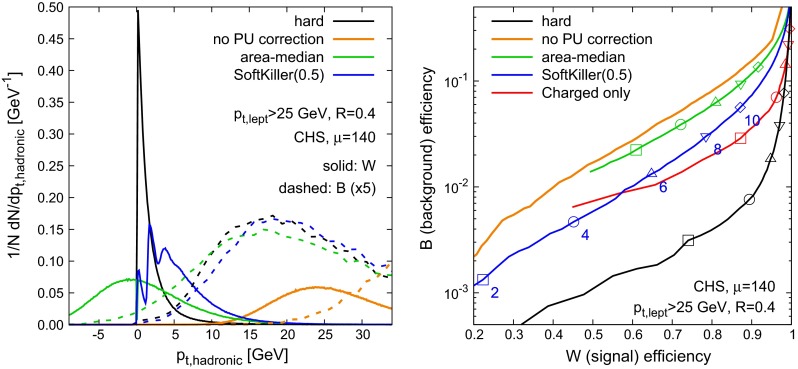
*Left* Simulated spectrum of (hadronic) $p_t$ in an $R=0.4$ cone around leptons with $p_t > 25~\text {GeV}$, for leptons from $W$ decay (*solid curves*) and from $B$-hadron decay (*dashed curves*); shown without pileup (*black curves*, “hard”) and with $\mu =140$ pileup together with various pileup mitigation approaches. *Right* Resulting ROC curves showing the efficiency of isolating leptons from $B$-hadrons versus leptons from $W$’s. The *different symbols* indicate specific choices of isolation $p_t$ cut (labelled in GeV for the *blue SK curve*)

## Appendix A: Collinear safety issues

Collinear safety is normally essential in order to get reliable results from perturbation theory. One reaction to the SoftKiller proposal is that it is not collinear safe, because it relies only on information about individual particles’ transverse momenta. There are at least two perspectives on why this is not a severe issue.

The first relates to the intrinsic low-$p_t$ nature of the $p_t^{\text {cut}}$, which is typically of order $1{-}2~\text {GeV}$. At these scales, non-perturbative dynamics effectively regulates the collinear divergence. Consider one element of the hadronisation process, namely resonance decay, specifically $\rho \rightarrow \pi \pi$: if the $\rho$ has a $p_t$ of order $2~\text {GeV}$, the rapidity-azimuth separation of the two pions is of the order of $0.7{-}1$ (see e.g. Ref. [14]). Alternatively, consider the emission from a high-energy parton of a gluon with a $p_t$ of the order of $1~\text {GeV}$: this gluon can only be considered perturbative if its transverse momentum relative to the emitter is at least of order a GeV, i.e. if it has an angle relative to the emitted of order $1$. Both these examples illustrate that the collinear divergence that is of concern at parton level is smeared by non-perturbative effects when considering low-$p_t$ particles. Furthermore, the impact of these effects on the jet $p_t$ will remain of the order of ${{p}_{t}^{\text {cut}}}$, i.e. power-suppressed with respect to the scale of hard physics.

The second perspective is from the strict point of view of perturbative calculations. One would not normally apply a pileup reduction mechanism in such a context. But it is conceivable that one might wish to *define* the final state such that it always includes a pileup and underlying event (UE) removal procedure.^17^ Then one should understand the consequences of applying the method at parton level. Considering patches of size $0.5 \times \pi /6$ and particles with $|y|<2.5$, there are a total of 120 patches; only when the perturbative calculation has at least $60$ particles, i.e. attains order $\alpha _s^{60}$, can $p_t^{\text {cut}}$ be non-zero; so the collinear safety issue would enter at an inconceivably high order, and all practical fixed-order parton-level calculations would give results that are unaffected by the procedure.

Collinear safety, as well as being important from a theoretical point of view, also has experimental relevance: for example, depending on its exact position, a particle may shower predominantly into one calorimeter tower or into two. Collinear safety helps ensure that results are independent of these details. While we carried out basic detector simulations in Sect. 4, a complete study of the impact of this type of effect would require full simulation and actual experimental reconstruction methods (e.g. particle flow or topoclustering).

## Appendix B: Rapidity dependence

One issue with the area–median method is that a global $\rho$ determination fails to account for the substantial rapidity dependence of the pileup contamination. Accordingly, the method is often extended by introducing an a priori determined function $f(y)$ that encodes the shape of the pileup’s dependence on rapidity $y$,

6 $$\begin{aligned} \rho (y) = f(y) \, \underset{i \in \mathrm{patches}}{\mathrm{median}} \left\{ \frac{p_{ti}}{A_i f(y_i)}\right\} . \end{aligned}$$

This is the approach that we have used throughout this paper.^18^ The rapidity dependence of $\rho$, shown as the dashed lines in Fig. 13 (left), is substantial and therefore we account for it through rescaling. The figure shows two different tunes (4C and Monash 2013), illustrating the fact that they have somewhat different rapidity dependence.

The SoftKiller method acts not on the average energy flow, but instead on the particle $p_t$’s. The solid lines in Fig. 13 (left) show that the average particle $p_t$ is nearly independent of rapidity. This suggests that there may not be a need to explicitly account for rapidity in the SK method, at least at particle level (detector effects introduce further non-trivial rapidity dependence).

This is confirmed in the right-hand plot of Fig. 13, which shows the rapidity dependence of the shift in the jet $p_t$ with the area–median and SK methods. Our default area–median curve, which includes rapidity rescaling, leads to a nearly rapidity-independent shift. However, without the rapidity rescaling, there are instead rapidity-dependent shifts of up to $10~\text {GeV}$ at high pileup. In contrast, the SK method, which in our implementation does not involve any parameterisation of rapidity dependence, automatically gives a jet $p_t$ shift that is fairly independent of rapidity, to within about $2~\text {GeV}$. We interpret this as a consequence of the fact (cf. the left-hand plot of Fig. 13) that the average particle $p_t$ appears to be far less dependent on rapidity than the average $p_t$ flow.^19^

## Appendix C: Monte Carlo tune dependence

While a full study of the dependence of the SK method on different Monte Carlo tunes is beyond the scope of this article, we have briefly verified that our conclusions are not affected by switching to another widely used LHC tune, the Pythia 6 [25] Z2 tune [26]. Figure 14 compares the Pythia 6 Z2 results for the jet $p_t$ offset and dispersion in a dijet sample with those from the Pythia 8 4C tune that we used throughout the article. While there are some differences between the two tunes, our main conclusions appear unchanged. In particular, the average $p_t$ shift remains under control, and there continues to be a significant improvement in the resolution.^20^

## Appendix D: Impact of a fixed $\varvec{p_t}$ cutoff

One key aspect of the SoftKiller approach is not simply that it applies a $p_t$ cutoff, but rather that there is a straightforward dynamical way of determining a $p_t$ cutoff, on an event-by-event basis, that removes the bulk of the effects of pileup with modest biases and improved dispersion.

For completeness, it is interesting to compare its performance to that of a fixed $p_t$ cut. Figure 15 shows the shifts (left) and dispersions (right), as a function of $n_\mathrm{PU}$, as obtained for the area–median method, the SoftKiller, and three fixed particle-level ${{p}_{t}^{\text {cut}}}$ values, $1$, $1.5$ and $2~\text {GeV}$. For each of these fixed ${{p}_{t}^{\text {cut}}}$ values, there is a value of $n_\text {PU}$ for which the shift in jet $p_t$ is zero, respectively $n_\text {PU}\simeq 20$, $60$ and $150$. However, as soon as one moves away from that particular $n_\text {PU}$ value, large biases appear. Around the $n_\text {PU}$ that has zero bias for a given fixed ${{p}_{t}^{\text {cut}}}$, the dispersion of the $p_t$ shift is quite close to that obtained in the SoftKiller approach. However, away from that $n_\text {PU}$ value, the dispersion becomes somewhat worse. Overall, therefore, the SoftKiller approach works noticeably better than any fixed cut.

One further study that we have carried out is to parametrise the average ${{p}_{t}^{\text {cut}}}$ shown in Fig. 2 (left) as a function of $n_\text {PU}$, and to apply a ${{p}_{t}^{\text {cut}}}$ that is chosen event-by-event according to that event’s actual value of $n_\text {PU}$. We have found that this has performance that is similar to that of the SoftKiller, i.e. SoftKiller’s slight event-by-event adaptation of the ${{p}_{t}^{\text {cut}}}$ for a fixed $n_\text {PU}$ [represented by the 1-$\sigma$ dashed lines in Fig. 2 (left)] does not appear to be critical to its success. This suggests that any approach that chooses an $n_\text {PU}$-dependent ${{p}_{t}^{\text {cut}}}$ so as to give a near-zero average $p_t$ shift may yield performance on dispersions that is similar to that of SoftKiller. From this point of view, SoftKiller provides an effective heuristic for the dynamic determination of the ${{p}_{t}^{\text {cut}}}$ value.

## Appendix E: Lepton isolation and (not) MET

Two non-jet-based quantities that suffer significantly from pileup effects are lepton isolation and missing transverse energy (MET) reconstruction.

Both potentially involve significant detector effects. For lepton isolation, we believe we may nevertheless be able to gain some insight by considering a simplified scenario. We consider isolation of hard leptons ($p_t > 25~\text {GeV}$) from $W$ decay and also of hard leptons (with the same $p_t$ cut) from $B$-hadron decays in events whose hard scattering was $gg$ or $q{\bar{q}} \rightarrow b{\bar{b}}$. The first sample provides genuinely isolated leptons, while the second provides a sample of non-primary leptons, i.e. one important source of lepton-production background that isolation is intended to eliminate. In both cases we use toy CHS events, as was described in Sect. 4.

Figure 16 (left) shows the $p_t$ contained in a cone of radius $0.4$ around the lepton, with solid curves for leptons from $W$’s and dashed curves for leptons from $B$ decays. All curves except the black one (hard event only, i.e. no pileup) correspond to events with a mean pileup of $\mu = 140$. The orange curves illustrate how pileup severely shifts and smears the distribution of $p_t$ around the lepton. Area–median subtraction eliminates the shift, but gives only a marginal improvement for the smearing. SK gives somewhat more improvement as concerns the smearing, but has the “feature” that there is a residual shift for the $W$ events, but not for the $B$-hadron decays. This difference is because $B$-hadron jets have some number of soft particles that are removed by SK, compensating for the small residual pileup left in by SK. In contrast leptons from $W$’s tend to have few genuine soft particles around them, so there is simply a net positive bias from the small leftover PU. The peaks in the SK $W$-sample curve correspond to having $0$, $1$, $2$, etc. residual pileup particles.

To establish how these characteristics translate to final performance, one should examine the ROC curve for “background” efficiency (i.e. for leptons from $B$’s) versus “signal” efficiency (i.e. leptons from $W$’s). These are shown in the right-hand plot of Fig. 16, with the symbols providing information about the isolation $p_t$ cut being used at a given point on the curve. Lower curves imply better performance. One sees that uncorrected pileup (orange curve) significantly degrades performance relative to the “hard” (i.e. no pileup) case. Area–median subtraction brings a small benefit and SK brings a further moderate improvement. For a given isolation $p_t$ cut, the area–median approach gives a relatively stable signal efficiency, while SK gives a more stable background efficiency.

A final comment about Fig. 16 (right) concerns the red curve, in which isolation is carried out just on the charged particles from the primary vertex. For signal efficiencies $\gtrsim 0.6$ this performs better than any pileup correction method (the exact value depends on the choice of $a$ for SK). This highlights the point that it may be better to discard pileup-contaminated information than it is to try to correct for the large impact of pileup.^21^ As well as discarding neutrals, one may also consider going to smaller isolation radii, keeping in mind also recent theoretical progress in understanding small-$R$ isolation and jets [48, 49]. The full optimisation over these various options should probably be left to detailed experimental work.

Let us finally briefly comment on MET. With a perfect, infinite acceptance detector, pileup would have almost no impact on MET, other than through the small fraction of neutrinos present in pileup. The large pileup-induced degradation in MET resolution that occurs in real life is almost entirely a result of the interplay between the detector (its acceptance and response) and pileup. Without a detailed full detector simulation, we believe that it is difficult for us to carry out a robust study of potential improvements in MET reconstruction with SK-inspired methods. Nevertheless, the fact that jet-area subtraction is used successfully in ATLAS MET reconstruction [50] suggests that the improvements from SK may be of benefit also for MET.

## Footnotes

1 One practically important aspect of the area–median method is the significant rapidity dependence of the pileup, most easily accounted for through a manually determined rapidity-dependent rescaling. This is discussed in detail in Appendix B.

2 Applying a $p_t$ threshold to individual particles is not collinear safe; in the specific context of pileup removal, we believe that this is not a significant issue, as we discuss in more detail in Appendix A.

3 For patch areas that are similar to the typical jet area, this can be expected to happen because half the patches will contain residual pileup of order ${{p}_{t}^{\text {cut}}}$, and since jets tend to have only a few low-$p_t$ particles from the hard scatter, the loss will also be order of ${{p}_{t}^{\text {cut}}}$.

4 In Appendix C we also briefly examine the Pythia 6 [25] Z2 tune [26], and find very similar results.

5 If one keeps the underlying event in the hard event, much of it (about $1~\text {GeV}$ for both the area–median approach and the SoftKiller) is subtracted together with the pileup correction, affecting slightly the observed shifts. Keeping massive particles does not affect the SK performance but requires an extra correction for the area–median subtraction [27]. We therefore use massless particles for simplicity.

6 For our purposes here, the version that we used is equivalent to the most recent public release, FastJet 3.0.6.

7 In a local parton–hadron duality type approach to calculate hadron spectra, the spectrum of very low $p_t$ particles in a jet of a given flavour is actually independent of the jet’s $p_t$ [31].

8 A study of fixed $p_t$ cutoffs, rather than dynamically determined ones, is performed in Appendix D.

9 Or $k_t$ jet radius.

10 In a context where the net shift is the sum of two opposite-sign sources of bias, this is perhaps not too surprising: the two contributions to the dispersion are each likely to be of the same order of magnitude as the individual biases, and their sum probably is to be minimised when neither bias is too large.

11 A single value of $a$ is adequate as long as jet finding is carried out mostly with jets of radius $R \simeq 0.4$. Later in this section we will supplement our $R=0.4$ studies with a discussion of larger jet radii.

12 Using a “safe” subtraction procedure that replaces negative-mass jets with zero-mass jets [14].

13 A further option is to use an “intrajet killer” that removes soft particles inside a given jet until a total $p_t$ of $\rho A_\mathrm{jet}$ has been subtracted. This shows performance similar to that of the Constituent Subtractor.

14 As an example, the $p_t$ threshold could be made to depend on a particle’s distance from the nearest jet core; however this then requires additional parameters to define what is meant by a nearby jet core and to parametrise the distance-dependence of the $p_t$ cut.

15 We use our standard choices for determining $\rho$, namely the grid version of the area–median method, with a grid spacing of $0.55$ and rapidity scaling as discussed in Appendix B. One could equally well use the same grid spacing for the $\rho$ determination as for the SoftKiller.

16 These timings have been obtained on an Intel Xeon processor, E5-2470 (2.20 GHz), using a development version of FastJet 3.1, with the “Best” clustering strategy. This has a speed that is similar to the public 3.0.6 version of FastJet. Significant speed improvements at high multiplicity are planned for inclusion in the public release of FastJet 3.1; however, they were not used here.
